# Co-circulation of Crimean–Congo hemorrhagic fever virus and spotted fever group *Rickettsia* in cattle and ticks from cattle in Kosovo

**DOI:** 10.3389/fvets.2026.1888406

**Published:** 2026-07-31

**Authors:** Jignesh Italiya, Kristýna Hrazdilová, Seyma S. Celina, Pavlina Nekudova, Tekkara Allan Obonyom, Avni Robaj, Jiri Cerny, David Modry, Lisa E. Hensley, Anne W. Rimoin, Ludek Zurek

**Affiliations:** 1 Department of Microbiology, Nutrition and Dietetics, Czech University of Life Sciences, Prague, Czechia; 2 Department of Chemistry and Biochemistry, Mendel University, Brno, Czechia; 3 Center for Infectious Animal Diseases, Faculty of Tropical AgriSciences, Czech University of Life Sciences Prague, Prague, Czechia; 4 National Agricultural Research Organization-Ngetta, Lira, Uganda; 5 Department of Veterinary Medicine, Faculty of Agriculture and Veterinary, University of Prishtina “Hasan Prishtina”, Prishtina, Kosovo; 6 Department of Veterinary Disciplines, Czech University of Life Sciences, Prague, Czechia; 7 Department of Botany and Zoology, Faculty of Science, Masaryk University, Brno, Czechia; 8 Institute of Parasitology, Biology Center of Czech Academy of Sciences, České Budějovice, Czechia; 9 Zoonotic Emerging Disease Research Unit, US Department of Agriculture, Manhattan, KS, United States; 10 Department of Epidemiology, UCLA Fielding School of Public Health, Los Angeles, CA, United States

**Keywords:** CCHFV, Crimean–Congo hemorrhagic fever virus, Kosovo, *Rhipicephalus annulatus*, *Rickettsia aeschlimannii*, SFG, tick-borne pathogens

## Abstract

**Background:**

Changes in climate and land use as well as expanding livestock production around the world, including southeastern Europe, result in growing public health risk of tick-borne pathogens. Cattle and other livestock serve as important hosts sustaining tick populations as well as tick-borne zoonotic and animal pathogens. This study aimed to investigate the prevalence and co-circulation of the spotted fever group (SFG) *Rickettsia* spp. in ticks from cattle and Crimean–Congo hemorrhagic fever virus (CCHFV) in cattle in Kosovo.

**Methods:**

Ticks from pastured cattle in the Malishevë district, Kosovo were screened for SFG *Rickettsia* spp. by polymerase chain reaction (PCR) and sequencing and cattle serum was screened for CCHFV antibodies by enzyme-linked immunosorbent assay (ELISA). Ticks were identified by amplification and sequencing of the partial *COI* gene.

**Results:**

All 110 ticks analyzed were identified as *Rhipicephalus annulatus.* SFG *Rickettsia* were detected in 9 ticks, seven *Rickettsia aeschlimannii* and two *Rickettsia sibirica* subsp. *mongolitimonae*, representing their first report in Kosovo as well as in *Rh. annulatus*. Serological assessment revealed CCHFV antibodies in 23 of 100 cattle indicating virus circulation.

**Conclusion:**

These findings demonstrate the simultaneous exposure of bacterial and viral tick-borne pathogens in a shared livestock interface. Integrated One Health surveillance is essential for early detection and risk mitigation in hyperendemic regions of the Western Balkans.

## Background

Tick-borne pathogens represent an increasing challenge across Eurasia, where climate change, land-use changes, and expanding livestock production intensify interactions among vectors, animals, and humans (1, 2). The complex ecological interactions in southeastern Europe heighten the epidemiological importance of cattle, which sustain large and diverse tick populations and serve as a host of several vector-borne zoonotic pathogens, including Crimean–Congo hemorrhagic fever virus (CCHFV) and spotted fever group (SFG) *Rickettsia* (3).

*Rickettsiae* are strict intracellular bacteria and are categorized into three major groups. Among these, the spotted fever group (SFG) *Rickettsia* represents a critical cluster of pathogens mainly transmitted to people by vectors such as ticks, fleas, and mites and are responsible for emerging zoonotic infections throughout Europe (4). Human SFG rickettsioses typically present as acute febrile illnesses characterized by fever, headache, rash, inoculation eschar, and regional lymphadenopathy, although clinical severity varies depending on the infecting *Rickettsia* species (5). In southeastern Europe and the Balkan peninsula, reported human infections are most frequently associated with *Rickettsia conorii*, while *Rickettsia aeschlimannii* and *Rickettsia slovaca* have increasingly been detected in ticks and sporadic human cases, highlighting their growing public health relevance. *Rickettsia aeschlimannii* is commonly associated with *Hyalomma* ticks and causes Mediterranean spotted fever-like illness, whereas *R. slovaca*, primarily transmitted by *Dermacentor* spp., is recognized as the causative agent of tick-borne lymphadenopathy (6–8). Tick activity and human exposure generally peak during the warmer spring and summer months (from April to September), correlating with the increased questing behavior of hard ticks with increased agricultural activities and livestock grazing (9). The emergence of these pathogens highlights their growing relevance to regional public health and underscores the need for enhanced active surveillance in Kosovo and the wider Western Balkan territory.

The epidemiology of CCHFV is well established, with Kosovo recognized as part of a broader endemic area characterized by virus circulation in humans, livestock, and ticks (10, 11). Serological and molecular studies have confirmed exposure in human and animal populations and phylogenetic analyses placed Kosovo strains within European lineages (12–14). The principal vectors and reservoirs of CCHFV are ticks of the genus *Hyalomma* that maintain enzootic cycles and enable spillovers to humans (12, 13). In contrast, knowledge of SFG *Rickettsia* in Kosovo remains limited. To date, molecular evidence is largely restricted to the detection of *Rickettsia conorii* subsp. *caspia* in *Rhipicephalus sanguineus* s.l., with few data available from livestock (15–17). *Rhipicephalus annulatus* is a one-host cattle tick principally recognized as a vector of veterinary pathogens such as *Babesia* spp. and *Anaplasma marginale*, and its involvement in SFG *Rickettsia* ecology remains poorly understood (18). Although diverse SFG *Rickettsia* species have been reported in neighboring countries (17), integrated studies assessing both bacterial and viral tick-borne pathogens within the same host - vector systems are lacking which limits understanding of their co-circulation in agro-pastoral environments.

In this context, the present study aimed to investigate the prevalence and phylogenetic relationships of SFG *Rickettsia* in ticks collected from cattle in Kosovo as well as to assess seroprevalence of cattle to CCHFV in the same localities, in order to evaluate the co-circulation of bacterial and viral tick-borne pathogens and their spatial overlap.

## Methods

### Study area and sampling design

Fieldwork was conducted in 2024 across 12 villages in the Malishevë district, Kosovo. A total of 110 adult ticks (106 partially engorged females and 4 males) parasitizing cattle and 100 bovine blood samples (Table 1) were collected by licensed veterinarians with farm-owner consent. Cattle and ticks originated from the same farms though not always from the same individual animals. The minimum sample size for this cross sectional study was estimated using Cochran’s sample size formula for prevalence studies: *n* = *Z*^2^*P*(1 − *P*)/*d*^2^, where n is the required sample size, *Z* = 1.96 (95% confidence level), *P* is the expected prevalence, and d is the desired absolute precision (19). For spotted fever group (SFG) *Rickettsia* spp., an expected prevalence of 16.2% based on previous data from Kosovo (5) and a precision of 7% yielded a minimum sample size of 107 ticks; therefore, 110 ticks were included. For CCHFV serology, an expected cattle seroprevalence of 19.21% (20) with an absolute precision of 7.7% yielded a minimum sample size of 100 cattle, matching the number of serum samples collected. Samples (ticks and blood) were transported to the laboratory at 4 °C and then kept frozen in −80 °C until further use.

**Table 1:** Prevalence of *Rickettsia* spp. in ticks from cattle and CCHFV-seropositive cattle across surveyed villages in Malishevë district (*confidence interval).

Village	Total ticks	*Rickettsia*-positive ticks	*Rickettsia* species	Total serum samples	CCHFV-positive cattle (%)	95% CI* (CCHFV)
Drenoc	21	2 (9.5%)	*R. aeshlimannii* *R. sibirica* subsp. *mongolitimonae*	12	0	0–24.2
Rud	4	0 (0%)	–	9	3 (33.3)	12.1–64.6
Damanek	8	3 (37.5%)	*R. aeshlimannii* (2) *R. sibirica* subsp. *mongolitimonae* (1)	6	4 (66.7)	30.0–90.3
Astrazup	12	1 (8.3%)	*R. aeshlimannii*	9	0	0–29.9
Turajkë	8	0 (0%)	–	12	7 (58.3)	31.9–80.7
Panorc	12	0 (0%)	–	10	5 (50.0)	23.7–76.3
Dragobil	13	2 (15.4%)	*R. aeshlimannii* (2)	9	2 (22.2)	6.3–54.7
Maxharre	4	0 (0%)	–	6	0	0–39.0
Qupev	8	0 (0%)	–	3	0	0–56.1
Bubel	8	1 (12.5%)	*R. aeshlimannii*	6	1 (16.7)	3.0–56.4
Lubizhdë	12	0 (0%)	–	12	1 (8.3)	1.5–35.4
Jançisht	0	–	–	6	0	0–39.0
Total	110	9 (8.2)	–	100	23 (23.0)	–

### Tick identification and DNA extraction

Ticks were identified morphologically using standard taxonomic keys (21). Each specimen was then bisected longitudinally; one half was homogenized, and genomic DNA was extracted using a commercial kit (Exgene™ Clinic SV Kit; GeneAll Biotechnology, Seoul, South Korea) with minor modifications as described by Daněk et al. (22). Tick species was confirmed by amplifying a 604 bp region of the mitochondrial *COI* gene using established primers (23). Amplicons were sequenced bidirectionally, and identities were verified via BLAST comparison.

### PCR detection of SFG Rickettsia

Samples were screened using PCR targeting partial *gltA*, *ompA*, and *ompB* genes. PCR was performed in a total volume of 25.0 μL using 2 × PCRBIO Taq Mix Red (PCR Biosystems, UK). Reaction conditions followed manufacturer instructions. Used primers and annealing temperatures are listed in Table 2. Amplicons of expected size were purified from gel using the GenePHlow Gel/PCR Kit (Geneaid Biotech Ltd., Taiwan) and Sanger sequenced using amplification primers (Macrogen Europe, Amsterdam, The Netherlands). Sequences were assembled and carefully edited in Geneious R11.1.5 software (24) and compared against GenBank entries.

**Table 2:** Primers and annealing temperatures used in this study.

Target gene		Name	Primers (5′ to >3′)	Product (bp)	Annealing (°C)	References
*gltA*	1st	Rick gltA_F2	TTCTGCATATGATGTTTGCAAC	550	56	This study
		Rick gltA_R1	ATTTGCGACGGTATACCCATAG			This study
	2nd	RpCS.780_new	GAYCATGAGCAGAAYGCTTCT	356	55	(54)
		Rick gltA_R3	TAGCTTCAAGTTCTATTGCTATTTG			This study
*ompA*	1st	Rr190.70p	ATGGCGAATATTTCTCCAAAA	630	53	(55)
		190.701r	GTTCCGTTAATGGCAGCAT			(56)
	2nd	ompA_FwIn	TCAAAAAGCAATACAACRAGG	580	52	This study
		ompA_RevIn	GACAGTTATTATACCTCCTCC			This study
*ompB*	1st	rompB_OR	GCTTTATAACCAGCTAAACCACC	1,311	55	(57)
		120–2788_new	AAACAATAATCAAGGTACTGTCAC			(58)
	2nd	ompB_FwIn	CAAGTAACGTTTACTACAGAC	1,182	50	This study
		ompB_RevIn	ACTTTGATGTGYATCAGTATAG			This study

### Phylogenetic analysis

All available *ompA* and *ompB* sequences of spotted fever group Rickettsia exceeding 500 bp and covering the amplified regions were downloaded from GenBank in March 2026 and analyzed separately for each gene; identical sequences were excluded. *Rickettsia felis* was included as an outgroup in both datasets. Open reading frames were unified prior to alignment. Sequences were aligned using a codon-based approach (transAlign) implemented in Geneious software, applying the bacterial genetic code (translation Table 2) and the MAFFT algorithm. Preliminary phylogenetic analyses (data not shown) were conducted using IQ-TREE (multicore version 2.1.3) (25), and the best-fit substitution models were selected automatically using ModelFinder according to the Bayesian Information Criterion (26).

For the final maximum-likelihood analysis, the dataset was reduced by selecting representative sequences for each clade to avoid redundancy, while all unique sequences obtained in this study were retained. The final alignments and phylogenies were constructed using the approach described above, and trees were reconstructed under the TVM + F + G4 model for *ompA* and the TVM + F + R2 model for *ompB*. Branch support was assessed using SH-aLRT and ultrafast bootstrap (UFBoot) (27, 28), each with 1,000 replicates.

The final *ompA* alignment comprised 113 sequences with 696 positions and 489 distinct patterns, whereas the *ompB* alignment included 79 sequences with 5,214 positions and 1,169 distinct patterns. Trees were visualized and edited in FigTree v1.4.4 and Inkscape 1.1.1.

### CCHFV serology

Cattle serum was separated from the blood samples (*n* = 100) by centrifugation and tested for anti-CCHFV antibodies using the ID Screen® CCHFV Double Antigen Multi-species ELISA (IDvet, France), following manufacturer instructions. ELISA results were validated using internal kit controls, and optical density values were interpreted according to the manufacturer’s recommended cut-off criteria.

## Results

A total of 110 adult ticks were collected from cattle herds in 11 villages of the Malishevë district. All specimens were identified morphologically as *Rhipicephalus annulatus* and the *COI* gene partial sequencing all 110 specimen confirmed this classification with 100% identity to GenBank reference sequences.

**Figure 1:**
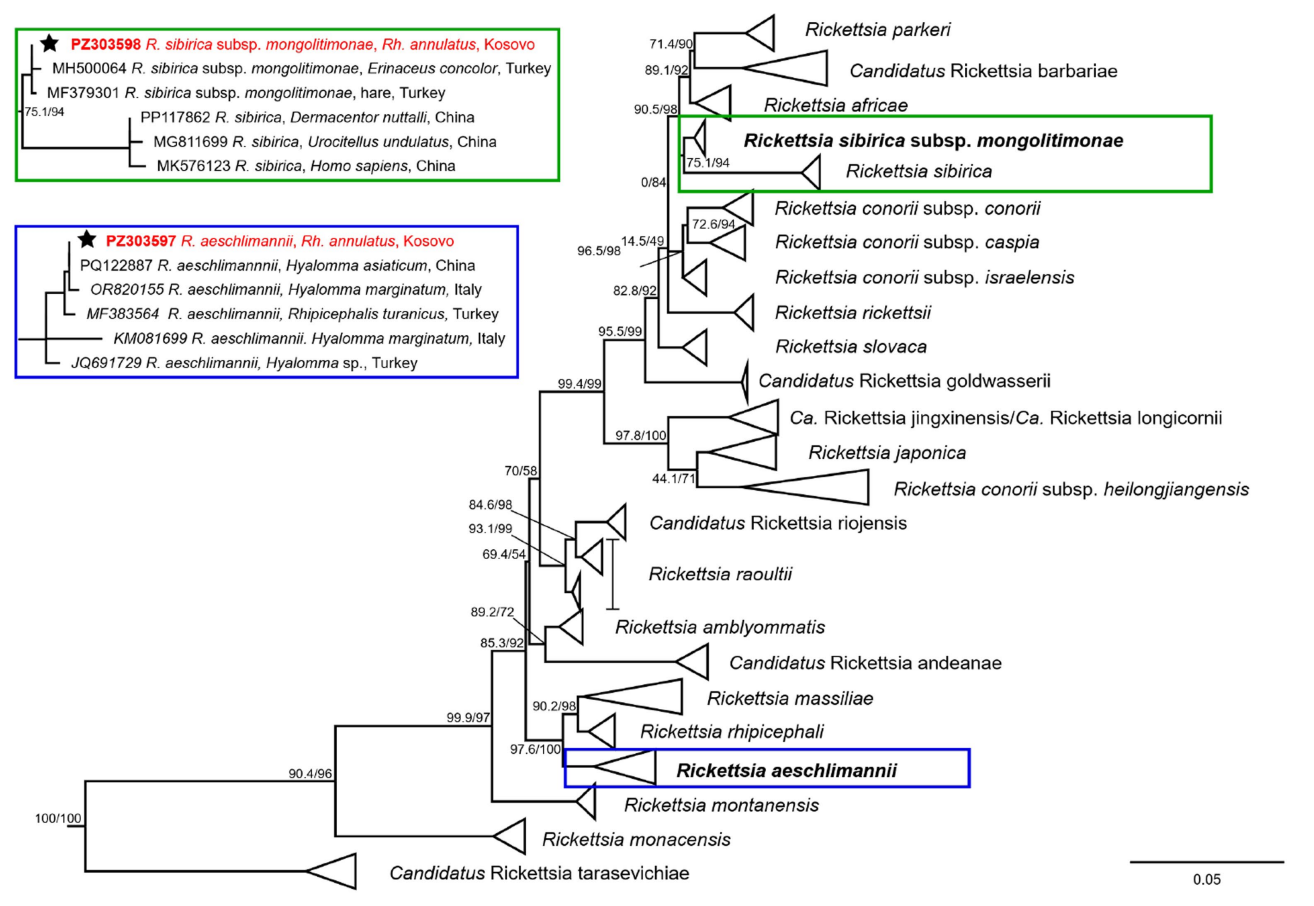
Simplified maximum-likelihood phylogenetic tree based on partial *ompA* sequences showing the position of Kosovo isolates. (Full version of the tree is available in Supplementary Figures S1, S2).

Screening for SFG *Rickettsia* revealed nine positive ticks (8.2%) from cattle herds in five villages, all confirmed by at least two gene targets (Table 1, Supplementary Table S1). Partial *gltA* sequences (~350 bp) were obtained for eight of nine positive samples and confirmed their assignment to *Rickettsia* spp., but were not used for species-level analyses due to their limited resolution. BLAST analysis of partial *ompA* and *ompB* sequences determined seven isolates as *Rickettsia aeschlimannii* originating from ticks from cattle in five villages, while the remaining two isolates showed the highest similarity to *Rickettsia sibirica* subsp. *mongolitimonae* from ticks from cattle from two different villages (Table 1). Phylogenetic analyses based on partial *ompA* and *ompB* sequences supported these findings. In the *ompA* analysis, Kosovo isolates of *R. aeschlimannii* clustered within a well-supported monophyletic clade of *R. aeschlimannii* sequences, sister to the *Rickettsia massiliae* and *Rickettsia rhipicephali* clades (Figure 1; Supplementary Figure S1). In the *ompB* analysis, the sequences obtained in this study also fell within the *R. aeschlimannii* clade, although with lower support (significant only in the SH-aLRT test), and were placed sister to *R. massiliae* and *Candidatus* Rickettsia shennongii (Supplementary Figure S2). In both the *ompA* and *ompB* trees, the two *Rickettsia sibirica* subsp. *mongolitimonae* sequences consistently formed a well-supported sister clade to *Rickettsia sibirica* lineages (Figure 1; Supplementary Figures S1, S2).

**Figure 2:**
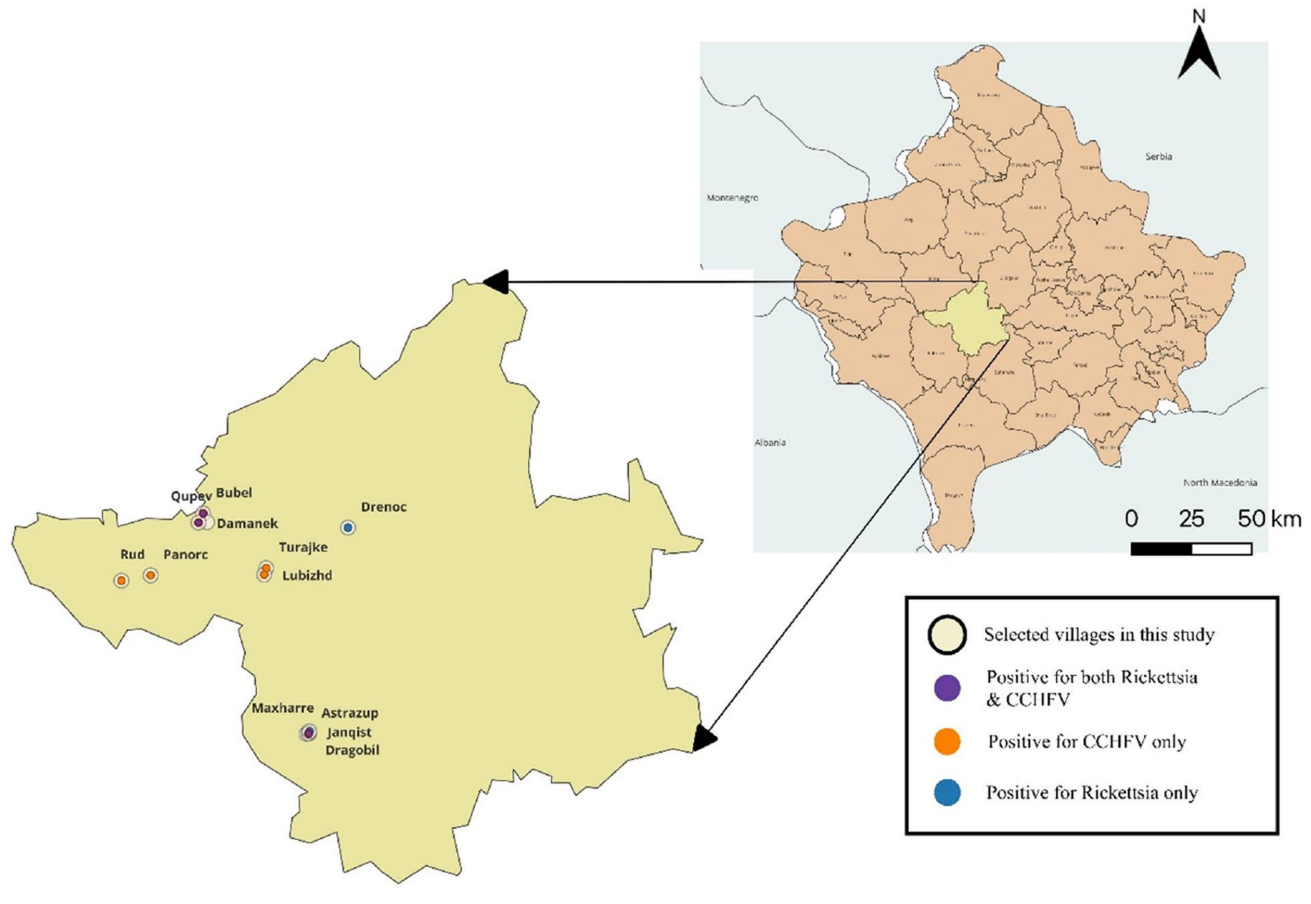
Spatial distribution of cattle from villages in Kosovo showing the detection of *Rickettsia* spp. in ticks and Crimean–Congo hemorrhagic fever virus (CCHFV) antibodies in cattle.

Serological testing of cattle revealed 23% CCHFV seropositivity (23/100), with positive animals distributed across eight villages (Table 1, Figure 2). Several sampled sites exhibited both, *Rickettsia*-positive ticks and CCHFV-seropositive cattle, demonstrating geographic overlap of bacterial and viral pathogen circulation within the same livestock herds (Table 1, Figure 2).

## Discussion

Tick-borne diseases represent an increasing One Health challenge across southeastern Europe, where environmental conditions, livestock management, and vector ecology converge to facilitate pathogen transmission. The Western Balkans, characterized by heterogeneous landscapes, traditional agro-pastoral practices, and favourable climatic conditions, supports large and diverse tick communities and therefore maintenance of multiple vector-borne zoonotic pathogens (29, 30). Within this system, cattle play a dual epidemiological role, sustaining large populations of livestock-feeding ticks and serving as a reservoir for pathogen circulation (31). This is particularly relevant for pathogens such as CCHFV and SFG *Rickettsia*, which co-exist within overlapping vector–host systems, primarily involving ticks of the genera *Hyalomma* and *Rhipicephalus* (3, 32, 33). The exclusive detection of *Rh. annulatus* in the present study likely reflects the focus on cattle-associated ticks in agro-pastoral systems, whereas previously reported *Rhipicephalus sanguineus* in Kosovo is primarily linked to dogs and peridomestic environments (33).

The 23% seroprevalence of CCHFV detected in cattle in this study confirms sustained viral circulation in Kosovo and is consistent with its long-standing classification as a hyperendemic region (34–36). Livestock plays a central role in CCHFV ecology, acting as amplifying hosts for ticks while remaining asymptomatic, making serological surveillance a good indicator of local transmission. And the persistence of competent vectors such as *Hyalomma marginatum* enables continued maintenance of the virus in the environment (3, 37).

The detection of *Rickettsia aeschlimannii* represents the first molecular evidence of this pathogen in Kosovo and expands its known distribution in southeastern Europe. This species is an emerging SFG *Rickettsia* widely reported across Africa, the Mediterranean basin, and parts of Asia, where it is primarily associated with *Hyalomma* ticks, particularly *Hyalomma marginatum* (38–41). Although *R. aeschlimannii* has occasionally been detected in some *Rhipicephalus* spp., such findings are relatively uncommon (42–45) and it has never been detected in *Rh. annulatus* previously and it is therefore novel and notable. Nevertheless, the presence of *R. aeschlimannii* in Kosovo is consistent with its established distribution across neighboring Serbia, North Macedonia, Greece and Turkey (46–48). Its spread has been linked to the expansion of *Hyalomma* ticks and long-distance dispersal via migratory birds transporting infected ticks (3, 49). In addition, regional livestock movements may facilitate the dissemination of infested ticks and contribute to the circulation of tick-borne pathogens across national borders. These mechanisms likely contribute to the introduction and silent maintenance of the pathogen in previously under-investigated areas and possible expansion of the vector range to *Rhipicephalus* spp.

The detection of *Rickettsia sibirica* subsp. *mongolitimonae* represents its first report in the country and further expands the diversity of SFG *Rickettsia* in Kosovo. This subspecies is a recognized human pathogen associated with Mediterranean spotted fever-like illness and has been documented across southern Europe, North Africa, and parts of Asia (8, 50–52). Similar to *R. aeschlimannii,* it is mainly vectored by *Hyalomma* ticks (39, 52, 53). Its detection in *Rh. annulatus* suggests either incidental acquisition or a broader ecological network of pathogen circulation involving multiple tick species. Alternatively, because all ticks analyzed in the present study were collected while feeding on cattle, the detected DNA may also reflect transient acquisition of pathogens from an infected blood meal rather than true infection of the tick. Since cattle blood samples were not screened for SFG *Rickettsia*, this possibility cannot be excluded and should be considered as a limitation of the present study. Published prevalences of both species in neighboring countries vary considerably among studies, largely because of differences in investigated tick species, host associations, geographical coverage, and molecular approaches, making direct quantitative comparisons difficult (6–8).

While the epidemiological significance of these new findings remains to be clarified, they highlight the need to reconsider simplified vector-pathogen relationships in favour of more integrated ecological models.

From the methodological perspective, the combined use of *gltA*, *ompA*, and *ompB* illustrates the complementary value of these markers for molecular surveillance of SFG *Rickettsia*. The highly conserved *gltA* gene is suitable for broad screening of SFG *Rickettsia*, but its limited sequence variability restricts species-level resolution. In contrast, the more variable *ompA* and *ompB* genes provide improved taxonomic discrimination and phylogenetic resolution, although amplification success may be lower because of sequence heterogeneity in primer-binding regions and the larger size of the target fragments (54–58).

Co-infestation of cattle by multiple tick species likely facilitates cross-species pathogen transfer through co-feeding or sequential feeding, suggesting that transmission networks of SFG *Rickettsia* are more complex than strict vector - pathogen associations imply (39, 43, 59). The observed geographic overlap between CCHFV-seropositive cattle and SFG *Rickettsia*-infected ticks further highlights the role of shared ecological drivers in shaping pathogen co-circulation. Factors such as extensive grazing, high host density, and climatic suitability promote both tick survival and pathogen transmission, creating localized microfoci where multiple zoonotic agents can persist (60, 61).

While this study provides foundational data on the co-circulation of CCHFV and SFG *Rickettsia* in livestock ticks interference in Kosovo, our findings should be interpreted within the context of a focused baseline investigation. It is important to stress out that CCHFV was detected in cattle by serology only whereas SFG *Rickettsia* sp. were detected by PCR from ticks only. Therefore, while our combined molecular and serological approach provides a valuable One Health perspective, future studies should analyze ticks and cattle by both approaches. In addition, the geographic scope, localized to the CCHFV hyperendemic Malishevë district, was intentionally selected to characterize this high-risk interface, and our results should be generalized to the wider Balkan region with caution. Larger longitudinal studies are needed to better understand the transmission of these zoonotic pathogens in the entire region.

In conclusion, the presence of CCHFV antibodies and multiple SFG *Rickettsia* species within the same livestock–tick systems illustrate the complexity of tick-borne pathogen circulation in Kosovo. These findings reinforce the importance of integrated One Health surveillance strategies that simultaneously address viral and bacterial pathogens across animal, vector, and human interfaces, particularly in regions with long-standing endemicity and favourable ecological conditions for vector persistence.

## Data Availability

The datasets presented in this study can be found in online repositories. The names of the repository/repositories and accession number(s) can be found at: https://www.ncbi.nlm.nih.gov/genbank/, PZ303598, PZ303597, PZ303602, PZ303601, PZ303600, and PZ303599.
