# Supplementary material for: Co-circulation of Crimean–Congo hemorrhagic fever virus and spotted fever group Rickettsia in cattle and ticks from cattle in Kosovo
Source: Front Vet Sci. 2026 Jul 31;13:1888406. doi: 10.3389/fvets.2026.1888406 (PMC13472809; doi:10.3389/fvets.2026.1888406)

Figure S2. Maximum-likelihood phylogenetic tree based on partial *ompB* sequences showing the position of Kosovo isolates.

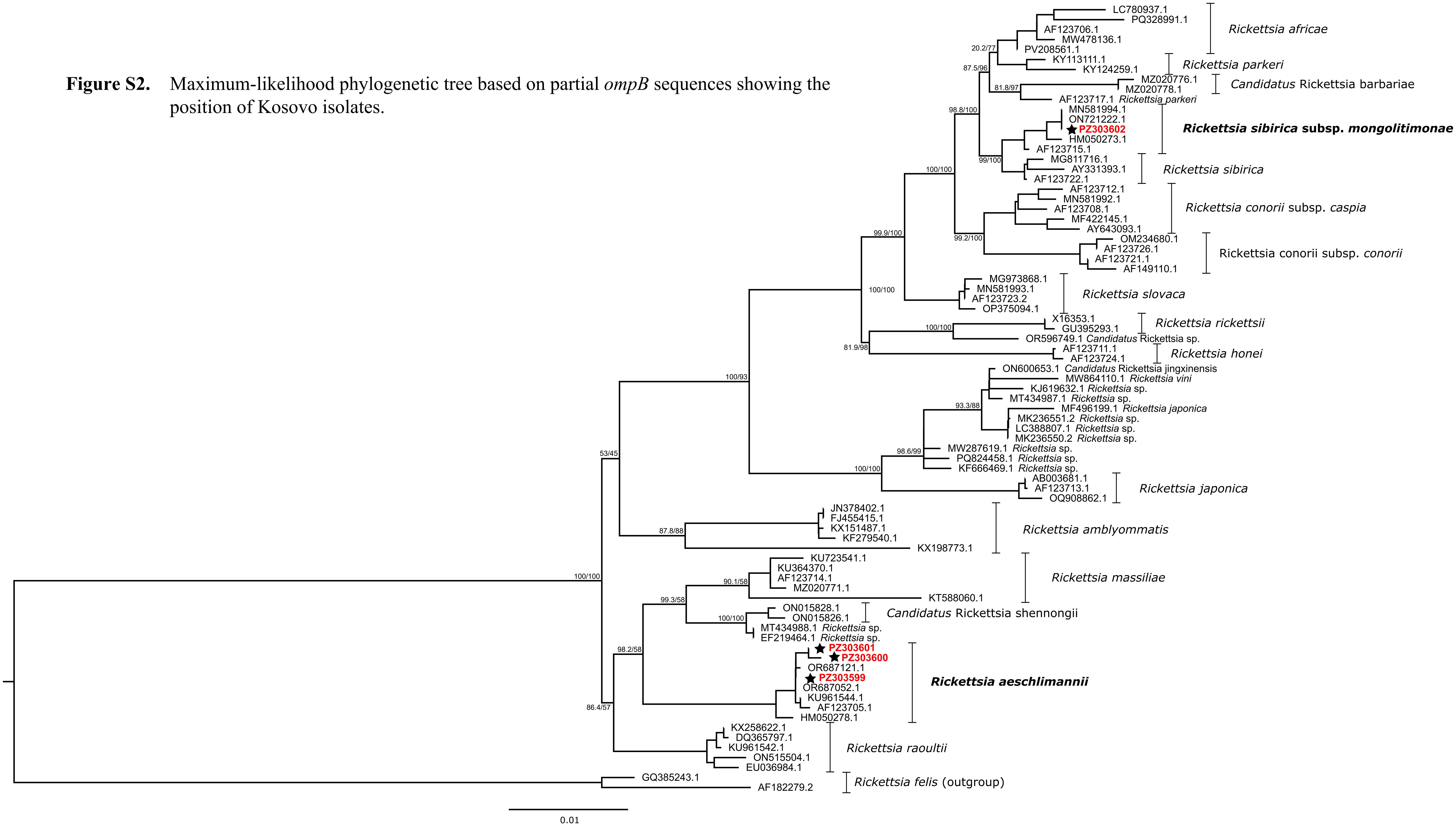

Supplement: Supplementary file 2 [file Image_2.pdf]
