# Supplementary material for: Co-circulation of Crimean–Congo hemorrhagic fever virus and spotted fever group Rickettsia in cattle and ticks from cattle in Kosovo
Source: Front Vet Sci. 2026 Jul 31;13:1888406. doi: 10.3389/fvets.2026.1888406 (PMC13472809; doi:10.3389/fvets.2026.1888406)
Supplement: Supplementary file 3 [file Table_1.docx]

**Table S1.** Identification of *Rickettsia* species based on genes *gltA, ompA, ompB*

| Sample no. | Species | *gltA* | *ompA* | *ompB* |
| --- | --- | --- | --- | --- |
| 27 | *R. aeschlimannii* | positive | n.a. | PZ303601 |
| 35 | *R. aeschlimannii* | positive | n.a. | PZ303601 |
| 57 | *R. aeschlimannii* | positive | PZ303597 | PZ303599 |
| 61 | *R. aeschlimannii* | positive | PZ303597 | PZ303600 |
| 63 | *R. sibirica* subsp. *mongolitimonae* | positive | n.a. | PZ303602 |
| 64 | *R. aeschlimannii* | positive | n.a. | PZ303601 |
| 89 | *R. aeschlimannii* | positive | PZ303597 | PZ303601 |
| 90 | *R. sibirica* subsp. *mongolitimonae* | n.a. | PZ303598 | PZ303602 |
| 110 | *R. aeschlimannii* | positive | PZ303597 | n.a. |

n.a. = not amplified
